# Measuring social capital of healthcare organizations reported by employees for creating positive workplaces - validation of the SOCAPO-E instrument

**DOI:** 10.1186/s12913-020-05105-9

**Published:** 2020-03-31

**Authors:** Lena Ansmann, Kira Isabel Hower, Markus Antonius Wirtz, Christoph Kowalski, Nicole Ernstmann, Lorna McKee, Holger Pfaff

**Affiliations:** 1 Division for Organizational Health Services Research, Department of Health Services Research, School of Medicine and Health Sciences, Carl von Ossietzky University Oldenburg, Ammerländer Heerstraße 140, 26129 Oldenburg, Germany; 2 Institute for Medical Sociology, Health Services Research and Rehabilitation Science, Faculty of Human Sciences and Faculty of Medicine, University of Cologne, Cologne, Germany; 3 Institute of Psychology, University of Education Freiburg, Freiburg, Germany; 4 German Cancer Society, Berlin, Germany; 5 Center for Health Communication and Health Services Research, Department of Psychosomatic Medicine and Psychotherapy, University Hospital Bonn, Bonn, Germany; 6 Health Services Research Unit, University of Aberdeen, Aberdeen, Scotland

**Keywords:** Social capital, Validation, Structural equation modelling, Work environment, Organization, Community, Burnout, Work engagement, Well-being

## Abstract

**Background:**

In highly segmented and complex healthcare organizations social capital is assumed to be of high relevance for the coordination of tasks in healthcare. So far, comprehensively validated instruments on social capital in healthcare organizations are lacking. The aim of this work is to validate an instrument measuring social capital in healthcare organizations.

**Methods:**

This validation study is based on a cross-sectional survey of 1050 hospital employees from 49 German hospitals which specialize in breast cancer care. Social capital was assessed by a six-item scale. Reliability analyses and confirmatory factor analyses were conducted to determine the content validity of items within the theory-driven one-dimensional scale structure. The scale’s associations with measures of the social aspects of the work environment (identification, social support, open communication climate) were estimated to test convergent validity. Criterion-related validity was evaluated by conducting structural equation modelling to examine the predictive validity of the scale with measures of work engagement, well-being and burnout.

**Results:**

A one-dimensional structure of the instrument could be identified (CFI = .99; RMSEA = .06). Convergent validity was shown by hypothesis-consistent correlations with social support offered by supervisors and colleagues, a climate of open communication, and employee commitment to the organization. Criterion-related validity of the social capital scale was proved by its prediction of employee work engagement (*R*^2^ = .10–.13 for the three subscales), well-being (R^2^ = .13), and burnout (*R*^2^ = .06–.11 for the three subscales).

**Conclusions:**

The confirmed associations between social capital and work engagement, burnout as well as well-being stress the importance of social capital as a vital resource for employee health and performance in healthcare organizations. In healthcare organizations this short instrument can be used as an efficient instrument to measure the organizations’ social capital.

## Background

Since the 1990s, healthcare organizations in modern healthcare systems experienced a constantly changing environment characterized among others by increasing economic pressure, restructuring and shortage of qualified healthcare professionals [1]. Healthcare organizations and their professionals thus perceived high insecurity [2, 3]. At the same time, healthcare became increasingly complex and specialized, which led to heterogeneous evolving professional identities and partly to fragmentation of healthcare, despite efforts towards integrated and managed care [4–6]. Highly-fragmented healthcare organizations often have deficits in the coordination of tasks such as inefficient communication between providers and with patients. Deficits in care coordination have comprehensively been described in cancer care and other diseases and associations with patient outcomes such as higher mortality have been shown [7, 8]. Therefore, achieving effective coordination of patient care has been declared by the Institute of Medicine [9] as a major challenge for healthcare organizations. Furthermore, high staff turnover and staff shortage is an upcoming problem in healthcare organizations. One proposed solution to solve both problems is to increase the social capital of employees working in healthcare organizations [10, 11]. The reason to propose the improvement of social capital as a solution to the problems mentioned is that it has two main functions: 1) for the organization: a performance-enhancing function [12], and 2) for the individual: a well-being function [13].

Theoretical arguments and empirical evidence foster the hypothesis that social capital is a latent resource enhancing organizational performance [12]. Especially, it fosters coordination and collective action [14]. Studies show that in healthcare organizations social capital fosters for example, quality and risk management [15–17] and quality of care [18–20]. A second function of social capital is to improve well-being and satisfaction at work, to create a sense of shared identity and belonging and thus diminish staff turnover and early retirement, also shown in healthcare organizations [13, 21–25]. The substantial body of evidence underlines the importance of social capital as a resource of healthcare organizations.

### Social capital: concepts and definitions

Social capital has been defined in many different ways, but there are two main schools of thought. The first is influenced by Coleman and Putnam. Putnam refers to social capital as “features of social organizations such as networks, norms and social trust that facilitate coordination and cooperation for mutual benefit” ([26], p., 69) and thus conceives it as a resource of the organization. The second main school of thought goes back to the work of Bourdieu, who defined social capital as “the aggregate of the actual or potential resources which are linked to possession of a durable network of more or less institutionalised relationships of mutual acquaintance and recognition” ([27], p., 248) and thus focuses more on resources that accrue to the individual as a result of social networks. For organizational research, Putnam’s definition is more appropriate, since he regards social capital as an organizational and not solely an individual resource [28]. In the literature different types and forms of social capital have been proposed. Bauman [29] together with other authors [14] brought up the term “community” as an integrative feature of collectivity that enables collective action by bringing people together. Bauman [29] describes ‘liquid modernity’ as characterized by high insecurity and constant change and at the same time individualization and segmentation of society. In those times people increasingly seek community to find mutual understanding, warmth, trust, a ‘we-feeling’, mutual help and shared values. Similarly, organizations increasingly have to act in an insecure and changing environment [30]. Thus, it can be assumed that their members seek community at the workplace and thereby build up social capital as an organizational resource [26]. Therefore, in the following the term ‘communal social capital’ is used to emphasize community’s social capital-building function. In highly segmented and complex healthcare organizations we believe that communal social capital is of high relevance for the coordination of tasks in healthcare.

#### Measurement of social capital in healthcare organizations

Whereas validated questionnaires on social capital in neighbourhoods and communities do exist [31], there is a lack of psychometrically tested and internationally published instruments on workplace social capital specifically for use in healthcare organizations. In a study in the United states of America, researchers adapted the World Bank’s Social Capital Integrated Questionnaire to measure the specific nursing work environment including 44 items [32]. An existing validated instrument for measuring workplace social capital that has been used among others in healthcare organizations was also developed by Kouvonen et al. in a study within the Finnish public sector [33–35]. The 8 item scale measures different components of social capital at the workplace and thereby focuses on leadership support rather than on communal social capital aspects. It was applied and validated in a large sample of more than 32,000 registered nurses, teachers, practical nurses and cleaners in Finland for several years. The scale showed good internal consistency, construct validity with related constructs such as procedural justice and criterion-related validity in relation to self-rated poor health at both an individual and organizational level. However, the factorial validity of the items has not been examined (e.g. in a structural equation model) and the analyses did not comprehensively take into account the organizational context. Additionally, Japanese researchers developed another workplace social capital scale [36]. It consists of six items related to cognitive social capital and is partly build upon the Kouvonen scale excluding the supervisor support items. However, as yet a validation study has not been published where the communal aspects of social capital have been explicitly included.

#### The SOCAPO-E instrument

We aimed to validate a short instrument to measure communal social capital of healthcare organizations reported by employees (SOCAPO-E). We define communal social capital as a feature of a social system which enables social integration through normatively guaranteed consensus. Based on the concept of community of Bauman, communal social capital is characterized by at least six central dimensions: mutual understanding, warm circle, trust, ‘we-feeling’ (i.e. a sense of being one of a team), mutual help and shared values [29]. These dimensions enable persons to coordinate their activities in an implicit and efficient way and to develop a healthy social climate [14].

### Scale development and utilization

In 2001 we developed nine items to measure communal social capital. By cognitive pre-testing we optimized content validity and comprehensibility. Additionally, we conducted an exploratory factor analysis on the basis of a hospital employee survey in Germany (*n* = 1645) which revealed six items loading on one factor [37], which represent the SOCAPO-E scale. Every element of the Bauman concept of community is represented in these six items, which we called communal social-capital. The SOCAPO-E scale from then on has been used in a variety of studies within the healthcare sector including hospitals, cancer centers, hospital boards, nurses and private practices. In these studies, social capital in healthcare organizations was associated with diverse indicators and outcomes, such as job satisfaction [24], burnout [23, 25], quality and risk management [15–17], perceived quality of care [19, 20] and turnover [22]. Although face validity was proven by cognitive pretesting and content validity appears to be given by the items’ representation of the six elements of community by Bauman, the instrument has never been completely validated beyond exploratory factor analysis. Also, the organizational nature of social capital has not been represented in previous factor analyses. The aim of the present work is to comprehensively validate the SOCAPO-E instrument for measuring communal social capital of healthcare organizations reported by employees.

## Methods

### Study design and data collection

The results of this study are based on a secondary analysis of data from a cross-sectional postal survey of hospital employees, conducted in 2010. This data was regarded as excellently suitable for the validation given the statistical power, the number of healthcare organizations and the instruments used. The survey was conducted in order to evaluate the introduction of breast cancer centers in the German state of North Rhine-Westphalia (NRW). All 53 accredited breast cancer centers in NRW were invited to participate. Since 81% of the centers were collaborations between two or three hospitals in close proximity, these 53 centers encompassed 90 hospitals. Hospitals performed a median of 157 surgeries on newly-diagnosed breast cancer patients per year and 60% were academic teaching hospitals. All healthcare professionals involved in caring for breast cancer patients at the time of the survey were invited to participate. Employees without direct contact to breast cancer patients were excluded (e.g. management staff). Recruitment and data collection were conducted between November 2010 and March 2011. After each participating hospital provided a list of eligible employees, the postal survey has been sent to the employee’s professional address. The survey was designed with three contact attempts being made. All participating employees provided their informed consent. Confidentiality was secured by using prepaid return envelopes, which were sent back to the research team. The survey was conducted in 49 German hospitals and 1050 out of 2061 employees of different occupations and levels of seniority participated in the survey (total response rate 51%, for physicians 46%, for nurses 50%, other staff such as physiotherapists or social workers 57%) [38]. Per hospital an average of 21 employees participated in the survey with response rates varying by hospital between 17 and 100%. Table 1 displays the sample characteristics and the SOCAPO-E mean values. Physicians constituted the majority of the sample (*n* = 387, 37%), followed by nurses (*n* = 330, 31%) and other staff (*n* = 317, 30%). The median age of the respondents was 46 years and the proportion of female employees amounted to 79%.

**Table 1:** Occupation, sex and age of the study sample (*n* = 1050)

	Frequency (n)	Percentage (%)	SOCAPO-E (mean, ***n*** = 951)
**Occupation**			
Physicians	387	36.9	3.01
Nurses	330	31.4	3.01
Other	317	30.2	2.90
Missing	16	1.5	–
**Sex**			
Male	217	20.7	3.04
Female	833	79.3	2.96
Missing	0	0	–
**Age**			
< 29 years	76	7.2	2.95
30–39 years	198	18.9	2.99
40–49 years	391	37.2	2.96
50–59 years	318	30.3	2.98
≥ 60 years	41	3.9	3.21
Missing	26	2.5	–
**Total**	1050	100.0	2.98

Data were collected using the “Employee survey for centers” questionnaire (MAZE) [38], which is a German instrument modified for use in cancer centers with the help of focus groups and cognitive pretest interviews with breast cancer center employees.

### Measures

#### The SOCAPO-E instrument to measure the social capital of healthcare organizations

The six item instrument described above captures all elements of the Bauman concept of community [29]: warm circle, mutual understanding, trust, mutual help, common values and ‘we-feeling’. In the context of this study we replaced the term “healthcare organization” with “hospital” (see Table 2). The participants answered the items on a four-point Likert scale ranging from 1 “I strongly disagree” to 4 “I strongly agree” (Cronbachs alpha .93).

**Table 2:** Items of the SOCAPO-E instrument based on community elements of social capital and intraclass correlation coefficients (ICC). Response options: “I strongly disagree” (1), “I somewhat disagree” (2), “I somewhat agree” (3), “I strongly agree” (4)

Item	Community elements of social capital	Items of the SOCAPO-E instrument	ICC
Soccap1	Mutual understanding	In our hospital, there is unity and agreement.	0.059
Soccap2	Trust	In our hospital, we trust one another.	0.037
Soccap3	We-feeling	In our hospital, there is a “we feeling” among the employees.	0.068
Soccap4	Warm circle	In our hospital, the work climate is good.	0.061
Soccap5	Mutual help & reciprocity	In our hospital, the willingness to help one another is great.	0.076
Soccap6	Common values	In our hospital, we share many common values.	0.061

#### Social support from supervisors and colleagues

The scales ‘social support from supervisors’ and ‘social support from colleagues’, originating from the Institute for Social Research, University of Michigan [39] were translated for the German context [40, 41]. The scales capture the employee-perceived willingness of supervisors and colleagues to support employees or colleagues with their work-related issues by three items each (example item: How much do your colleagues support you so that it is easier for you at work?). Respondents have to choose one answer on a four-point Likert scale ranging from 1 “not at all” to 4 “completely” (Cronbachs alpha .90, .88).

#### Open communication

The scale ‘open communication’ has been developed and used in previous studies [37]. It captures the employee-perceived possibility at the workplace to comment openly on problems, to offer criticism and to participate in decision making processes (example item: In our workplace problems are openly discussed). Respondents are asked to answer four items on a four-point Likert scale ranging from 1 “I strongly disagree” to 4 “I strongly agree” (Cronbachs alpha .88).

#### Identification with the breast cancer center

This newly-developed scale measures the degree of identification with the healthcare organization, in this case the breast cancer center. Respondents are asked to answer five items on a seven-point Likert scale ranging from 1 “not at all” to 7 “very much” (Cronbachs alpha .84). An example item is “How strongly do you feel a shared responsibility for the breast cancer center’s success?”

#### Work engagement

The short version of the Utrecht Work Engagement Scale (UWES-9) is an internationally established and validated instrument, which is used in an authorized translated version in German [42]. The instrument assesses a positive work-related state of fulfillment that is characterized by vigor, dedication, and absorption, characterizing the three subscales (example item: At my job, I feel strong and vigorous). The subscales contain three items each to be answered on a seven-point Likert scale from 0 “never” to 6 “always” (Cronbachs alpha .76, .86, .87).

#### Burnout

The Maslach Burnout Inventory for Human Services (MBI-HSS) was utilized in an authorized German version adapted from Büssing [43]. The instrument consists of the three subscales emotional exhaustion (9 items), depersonalization (5 items) and personal accomplishment (7 items) (example item: I feel used up at the end of the workday). Employees were asked to answer on a six-point Likert scale from 1 “never” to 6 “very often” (Cronbachs alpha .90, .71, .79).

#### Well-being

Psychological well-being was measured by using the German version of the WHO-5, which is the most widely accepted instrument assessing subjective psychological well-being [44]. The WHO-5 instrument measures current mental well-being by five items (example item: I have felt calm and relaxed). Employees were asked to answer on a six-point Likert scale from 0 “not present” to 5 “constantly present” (Cronbachs alpha .89).

### Data analysis

Following the theoretical derivation, the social capital scale’s psychometric quality was evaluated following Kline’s [45] two-step procedure. For the first step, a confirmatory factor analysis (CFA) was conducted to test the one-dimensional structure by evaluating global and local fit indices. CFA is recommended to confirm factorial validity on scale and item -level. For the second step, bivariate analyses and multivariate structural equation modelling (SEM) were conducted to confirm the construct validity by using convergent validity and criterion-related validity. Given the clustered structure of the data (employees in hospitals), multilevel CFA was conducted (*type = twolevel* in Mplus). In the bivariate analyses and SEM, we accounted for the clustered structure by adjusting standard errors (*type = complex* in Mplus), assuming level invariant factor structures. Cases with missing data were excluded from the analyses. The maximum-likelihood estimation procedure [45] in Mplus 8 software was used to define and test CFA and SEM. SPSS Version 25 was used to compute descriptive statistics and manifest bivariate analysis.

#### Factorial validity

In the first step, factor loadings of the six items of the SOCAPO-E instrument were verified. Loadings ≤.71 were interpreted as excellent, ≤.63 as very good, ≤ 55 as good, ≤.45 as fair, and ≤ .32 as poor [45]. Local fit indices assess whether constructs can be reliably estimated from their indicators. Recommended thresholds were used to determine a good model fit: Average Variance Extracted (AVE) ≥ .5, factor reliability ≥.6, reliability (Cronbach’s Alpha) ≥ .7, Residual-Correlations (≤.3). Various global fit indices have to be met to accept the model as plausible and parsimonious. Several cut-off values for fit indices have been suggested to evaluate model fit: Root Mean Square Error of Approximation (RMSEA) ≤ .08 (acceptable), ≤.05 (good) and incremental fit indexes (Comparative Fit Index (CFI) and Tucker-Lewis Index (TLI) (≥.95: acceptable; ≥.97: good).

#### Convergent validity

We analyzed bivariate correlations of the SOCAPO-E instrument with theoretically related constructs of the social aspects of the work environment: social support from supervisors and colleagues, open communication, and identification with the breast center.

#### Criterion-related validity

The wider literature suggested that social capital predicts outcomes of performance and employee health in healthcare organizations (see background section). Therefore, the SOCAPO-E instrument was assumed to be associated with perceived work engagement, burnout, and well-being. Conducting SEM provided information about the predictive validity of the SOCAPO-E instrument in order to evaluate criterion-related validity.

## Results

### Content validity

**Fig. 1:**
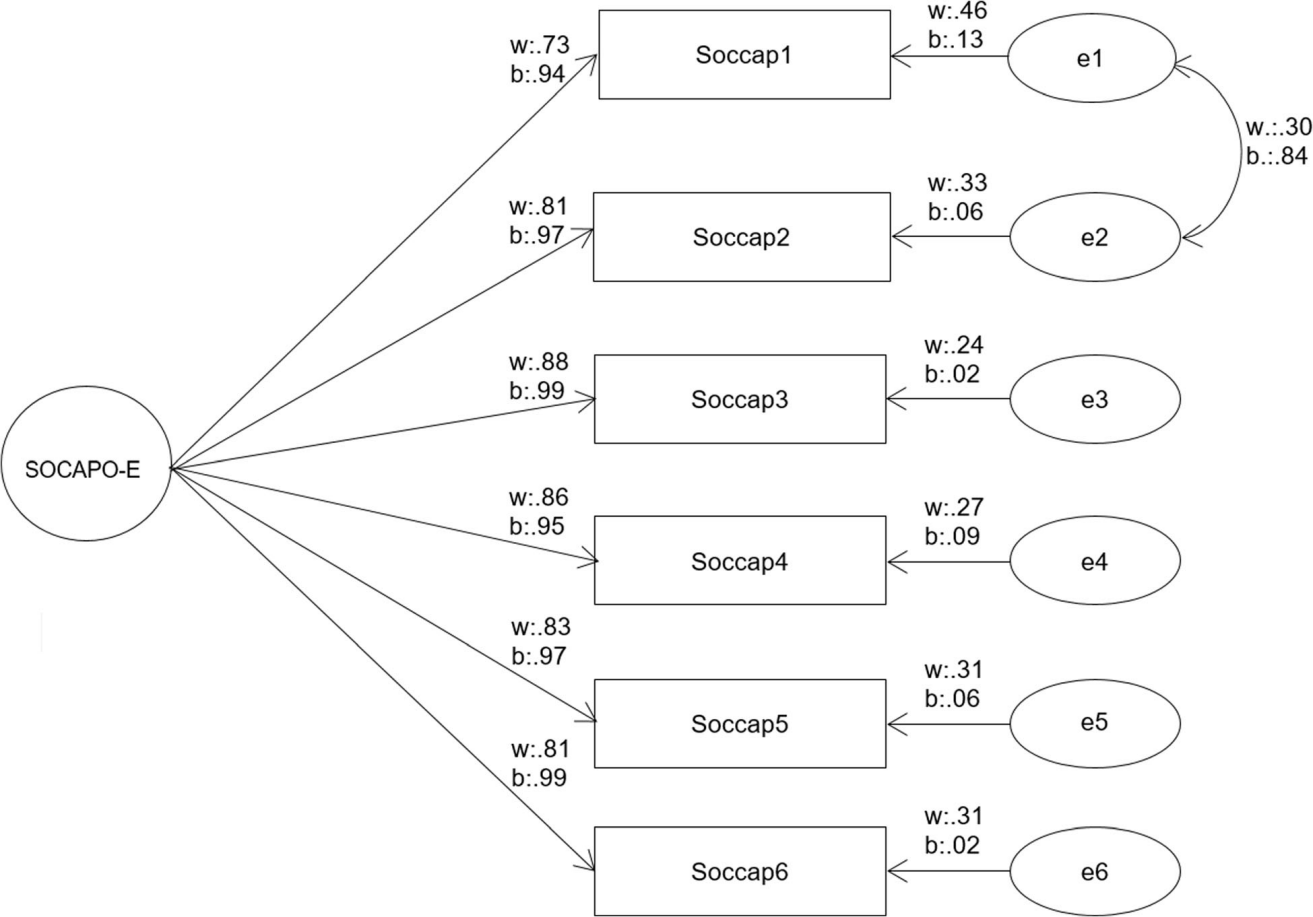
Confirmatory model of the SOCAPO-E instrument. Notification: w: within-level; b: between-level

Since *n* = 99 respondents had a missing on at least one of the six SOCAPO-E items, the number of analyzed responses reduced to *n* = 951. In the first step of the analysis, the one-dimensional structure of the SOCAPO-E instrument was tested using a multilevel confirmatory model (see Fig. 1). The six items displayed in Table 2 build the basis for this analysis. The mean SOCAPO-E score over all respondents was 2.98, whereas the hospital with the lowest score had a mean value of 2.47 (*n* = 37, response rate 45%) compared to a mean value of 3.41 (*n* = 30, response rate 51%) of the hospital with the highest score. The intraclass correlation coefficient (ICC), i.e. the proportion of variance in the items attributable to differences between hospitals, ranges between 0.037 and 0.076.

The measures of global fit are shown in Table 3. The indicators reveal that the original social capital model appears not to have an optimal incremental model adjustment. The RMSEA of .09 indicates moderate differences between data and model predictions. Analyzing the residual correlations revealed a substantial association of item one and two. This indicates that both items - mutual understanding and trust - correlated higher as a one-factorial model would suggest [45]. Consequently, their covariance was treated as a free parameter in the second model (modified social capital model, see Table 3). The new model (see Fig. 1) now shows a good fit as indicated by the parameters. The modification does not alter the associations between the variables and the factor in the SEM.

**Table 3:** Indicators of global model fit

	χ^**2**^	Df	TLI	CFI	RMSEA
**Threshold for acceptable fit**			≥.95	≥.95	≤.06
**Social capital model**	153.16	18	.95	.97	.09
**Modified social capital model (**Fig. 1**)**	73.95	16	.97	.99	.06

*χ*^*2*^ chi square, *Df* degrees of freedom

Local fit indices verified that the social capital construct is reliably measured by its indicators. In the modified model, all standardised factor loadings were significant (*p* < .001) and higher than .5. (see Fig. 1). The Average Variance Extracted was ≥.5 (AVE = .75). Reliability measures (Cronbach’s Alpha (.93), factor reliability (.95), and residual correlations (. ≤ .3)) comply with critical values and indicated that the measure is reliable (see Table 4 for residual correlations on individual and organizational level).

**Table 4:** Empirical residual correlation matrix of the six items of the SOCAPO-E instrument

Items	Soccap1	Soccap2	Soccap3	Soccap4	Soccap5	Soccap6
*Residual correlations within (individual level)*						
Soccap1	-					
Soccap2	.000	.000				
Soccap3	.013	.021	.000			
Soccap4	.015	.009	.000	.000		
Soccap5	−.018	−.014	−.025	.003	.000	
Soccap6	−.018	−.028	.003	−.018	.050	.000
*Residual correlations between (organizational level)*						
Soccap1	-					
Soccap2	−.005	.000				
Soccap3	−.018	−.006	.000			
Soccap4	.081	.069	−.002	.000		
Soccap5	−.035	−.030	.003	−.025	.000	
Soccap6	−.003	−.032	−.007	−.028	.010	.000

### Convergent validity

We analyzed bivariate correlations with “social support from supervisors”, “social support from colleagues”, “open communication”, and “identification with the breast center“ in an overall model including all measures and in separate models with each measure independently (see Table 5). The SOCAPO-E instrument correlated significantly (*p* ≤ .01) with all measures of the social aspects of the work environment both in the full model and in the bivariate analyses: social support from colleagues (SUC) and supervisors (SUS), open communication (OC), and identification with the breast center (IDE).

**Table 5:** Bivariate and full models of measurement quality (factor loadings and global fit indices) of measures of the social aspects of the work environment and correlations with the SOCAPO-E instrument. SUS: social support by supervisors, SUC: social support by colleagues, OC: open communication, IDE: identification with the breast cancer center

		Bivariate Models							Full Model						
		Estimate	S.E.	*p*-value	SRMR	RMSEA	CFI	TLI	Estimate	S.E.	*p*-value	SRMR	RMSEA	CFI	TLI
SUS	SUV1	0.829	0.013	< 0.01	0.018	0.041	0.993	0.990	0.831	0.013	< 0.01	0.026	0.032	0.984	0.981
	SUV2	0.897	0.011	< 0.01					0.894	0.012	< 0.01				
	SUV3	0.855	0.017	< 0.01					0.855	0.017	< 0.01				
	Corr	0.590	0.028	< 0.01					0.592	0.028	< 0.01				
SUC	SUK1	0.788	0.018	< 0.01	0.018	0.042	0.992	0.989	0.789	0.017	< 0.01				
	SUK2	0.886	0.016	< 0.01					0.885	0.015	< 0.01				
	SUK3	0.864	0.018	< 0.01					0.865	0.017	< 0.01				
	Corr	0.594	0.029	< 0.01					0.593	0.029	< 0.01				
OC	OVK1	0.848	0.013	< 0.01	0.024	0.039	0.992	0.988	0.846	0.013	< 0.01				
	OVK2	0.863	0.013	< 0.01					0.867	0.013	< 0.01				
	OVK3	0.773	0.018	< 0.01					0.772	0.019	< 0.01				
	OVK4	0.725	0.023	< 0.01					0.725	0.023	< 0.01				
	Corr	0.677	0.027	< 0.01					0.673	0.027	< 0.01				
IDE	INT1	0.875	0.023	< 0.01	0.024	0.038	0.990	0.986	0.873	0.024	< 0.01				
	INT2	0.874	0.017	< 0.01					0.877	0.017	< 0.01				
	INT3	0.555	0.031	< 0.01					0.554	0.032	< 0.01				
	INT4*	0.603	0.031	< 0.01					0.603	0.032	< 0.01				
	INT5*	0.684	0.023	< 0.01					0.684	0.023	< 0.01				
	Corr	0.466	0.035	< 0.01					0.445	0.035	< 0.01				

### Criterion-related validity

Conducting SEM verified the predictive validity of the SOCAPO-E instrument (see Table 6). The estimations showed significant paths from SOCAPO-E to the three subscales of “work engagement”: subscale “vigor” (VIG), subscale “dedication” (DED), subscale “absorption” (ABS) as well as to the three subscales of “burnout”: subscale “emotional exhaustion” (EE), subscale “depersonalisation” (DEP), subscale “personal accomplishment” (PER); and “well-being” (WHO5).

**Table 6:** Bivariate and full models of associations from the SOCAPO-E instrument on outcomes of performance and health. VIG: work engagement – vigor, DED: work engagement – dedication, ABS: work engagement – absorption, EE: burnout – emotional exhaustion, DEP: burnout – depersonalization, PER: burnout – personal accomplishment, WHO5 – well-being

Bivariate Models								Full Model					
SOCAPO-E instrument correlated with	Beta coefficient (S.E.)	*p*-value	R^2^	SRMR	RMSEA	CFI	TLI	Beta coefficient (S.E.)	*p*-value	SRMR	RMSEA	CFI	TLI
VIG	.348 (.043)	< 0.01	.126	.031	.064	.977	.968	.355 (.040)	< 0.01	.053	.895	.885	.053
DED	.318 (.043)	< 0.01	.104	.026	.064	.977	.969	.322 (.043)	< 0.01				
ABS	.305 (.043)	< 0.01	.095	.027	.060	.981	.974	.309 (.041)	< 0.01				
EE	−.340 (.038)	< 0.01	.110	.040	.066	.949	.939	−.331 (.039)	< 0.01				
DEP	−.244 (.040)	< 0.01	.063	.028	.052	.973	.966	−.251 (.040)	< 0.01				
PER	−.299 (.056)	< 0.01	.085	. 046	. 054	. 959	. 950	−.291 (.057)	< 0.01				
WHO5	.359 (.039)	< 0.01	.128	.026	.065	.970	.962	358 (.040)	< 0.01				

## Discussion

The aim of this validation study was to examine the reliability and validity of an instrument to measure communal social capital in healthcare organizations.

The results show that the SOCAPO-E instrument can adequately be modelled using a six-item solution and one modification. In factor analysis, modifications are common and reflect correlations between particular items of the instrument, however these correlations are to be expected within items of a construct. The modification was applied to statistically consider these correlations, but does not change the theoretical understanding of the model, in which the six items reflect the six elements of community by Bauman [29]. The global fit indices as well as the local fit indices underlined the unidimensional representation of the instrument. The determined correlations with instruments measuring similar constructs (identification, social support by supervisors and colleagues, open communication) are indicators of the convergent validity of the SOCAPO-E instrument. Nevertheless, correlations show that the construct can be differentiated from these similar constructs. Furthermore, the SEM analyzing criterion-based validity shows that the social capital construct predicts aspects of employee performance in terms of work engagement and employee health in terms of well-being and burnout. These results align with previous studies showing associations between the SOCAPO-E instrument and burnout [20, 25]. Moreover, the instrument used by Kouvonen et al. 2006 [33] has shown associations with self-rated health, which aligns with our results regarding associations with well-being. Thus, the instrument is able to predict associations to relevant outcomes.

### Strengths and limitations

The findings presented have to be considered in the light of methodological limitations. The secondary data from 2010 probably does not reflect recent trends in healthcare. However, we believe that the associations found are rather basic and stable relationships, which only vary by time in regard to their strength. We are also confident that the understanding of the items of the SOCAPO-E instrument itself is still largely the same compared to today. Furthermore, the cross-sectional study design does neither allows causal conclusions nor conclusions about the sensitivity of change of the SOCAPO-E instrument. Moreover, the instruments measuring open communication and identification with the breast cancer center, which have been utilized for convergent and criterion-related validity, have previously been validated by exploratory factor analysis and reliability analysis, but not by CFA. However, the validity and reliability analyses presented have confirmed their psychometric quality with one modification. The modification did not lead to a change in the theoretical understanding of the model. Since all investigated scales stem from the same survey, an overestimation of the explanation of variance may be possible due to common method bias. Unfortunately, the survey data could not be matched with independently collected criterion measures. The multilevel approach in the present analyses takes into account the hierarchical data structure within the SEM and the fact that workplace social capital is a construct at an organizational rather than at an individual level. This is only possible with the present large dataset of employees in 49 hospitals with a good response rate. Although the results are representative specifically for breast cancer care in the German state of North Rhine-Westphalia, the SOCAPO-E instrument has already been successfully applied in many different healthcare settings including hospital boards [11, 15], wards [19, 23] as well as private practices [22] in Germany and internationally [20, 46, 47]. Thus, the applicability to different types of healthcare organizations outside of breast cancer care can be assumed. In the present sample, female healthcare professionals represented the clear majority as to be expected in the healthcare sector. However, since the data showed on average slightly lower social capital for women compared to men, this sex difference should further be investigated. The instrument has already been translated and used in English as well as in a Dutch and French version in hospitals in Belgium [48]. However, the applicability should further be proven by validations in different contexts and languages. In addition, it is a short instrument that can efficiently be included into employee surveys.

### Implications for research and practice

A few studies already suggest associations between social capital and patient care, such as the healthcare organizations’ implementation of quality and risk management [15–17], quality of care perceived by healthcare professionals [18, 20] and the patient-physician relationship as perceived by patients [19]. To gain a better understanding of the relevance of communal social capital for patients, associations between social capital of healthcare organizations and patient outcomes should be studied. In addition, the SOCAPO-E instrument could be used in future studies in order to test interventions to facilitate social capital in healthcare organizations. Research on social capital yields approaches by which social capital in organizations can be strengthened [10, 11]. Approaches embrace (1) reasoned staff selection with focus on social skills, cooperative behaviour and trustworthiness, (2) enabling opportunities to meet face-to-face (e.g. weekly meetings, social events) and thereby developing social ties between employees, and (3) mentoring programs for new employees to convey the organization’s norms. (4) Lastly, a randomized controlled trial found group-based physical exercise at work to be effective for fostering social capital in hospitals [49].

## Conclusions

The SOCAPO-E instrument represents a short, valid and theory-based instrument to measure the communal social capital of healthcare organizations. It can be used as a valid instrument in studies focusing on the effects and associations of social capital and potentially also for evaluating interventions facilitating social capital of organizations, whereas the sensitivity to change still has to be shown. Particularly, the confirmed associations between social capital and work engagement, burnout as well as well-being stress the importance of social capital as a vital resource for employee health and performance in healthcare organizations.

## Data Availability

The datasets used and/or analyzed during the current study are available from the corresponding author on reasonable request.

## Abbreviations

ABS: Absorption
AVE: Average Variance Extracted
CFA: Confirmatory factor analysis
CFI: Comparative fit index
DED: Dedication
DEP: Depersonalization
EE: Emotional exhaustion
ICC: Intraclass correlation coefficient
IDE: Identification with the breast center
MAZE: Employee survey for centers
MBI-HSS: Maslach Burnout Inventory for Human Services
OC: Open communication
PER: Personal accomplishment
RMSEA: Root Mean Square Error of Approximation
SEM: Structural Equation Modeling
SOCAPO-E: Communal Social Capital of Healthcare Organizations Reported by Employees
SUC: Social support from colleagues
SUS: Social support from supervisors
TLI: Tucker-Lewis Index
UWES-9: Utrecht Work Engagement Scale-9
VIG: Vigor
WHO-5: WHO-Five Well-being Index
WHO5: Wellbeing

## References

[1] Scheffler RM, Arnold DR (2019). Projecting shortages and surpluses of doctors and nurses in the OECD: what looms ahead. Health Econ Policy Law..

[2] Ostry AS, Spiegel JM (2004). Labor markets and employment insecurity: impacts of globalization on service and healthcare-sector workforces. Int J Occup Environ Health.

[3] Burke RJ, Ng ESW, Wolpin J (2015). Economic austerity and healthcare restructuring: correlates and consequences of nursing job insecurity. Int J Hum Resource Manag.

[4] Gittell JH, Weiss L (2004). Coordination networks within and across organizations: a multi-level framework. J Management Studs.

[5] Cebul RD, Rebitzer JB, Taylor LJ, Votruba ME (2008). Organizational fragmentation and care quality in the U. S healthcare system. J Econ Perspect.

[6] Nolte E, Knai C, Hofmarcher M, Conklin A, Erler A, Elissen A (2012). Overcoming fragmentation in health care: chronic care in Austria, Germany and the Netherlands. Health Econ Policy Law.

[7] Gorin SS, Haggstrom D, Han PKJ, Fairfield KM, Krebs P, Clauser SB (2017). Cancer care coordination: a systematic review and meta-analysis of over 30 years of empirical studies. Ann Behav Med.

[8] Snow K, Galaviz K, Turbow S. Patient outcomes following interhospital care fragmentation: a systematic review. J Gen Intern Med. 2019. 10.1007/s11606-019-05366-z. 10.1007/s11606-019-05366-z PMC7210367 31625038

[9] Institute of Medicine (2001). Crossing the quality chasm: a new health system for the 21^st^ century. Washington (DC).

[10] Hofmeyer A, Marck PB (2008). Building social capital in healthcare organizations: thinking ecologically for safer care. Nurs Outlook.

[11] Gloede TD, Hammer A, Ommen O, Ernstmann N, Pfaff H (2013). Is social capital as perceived by the medical director associated with coordination among hospital staff? A nationwide survey in German hospitals. J Interprof Care.

[12] Jiang JY, Liu C-W (2015). High performance work systems and organizational effectiveness: the mediating role of social capital. Hum Resour Manag Rev.

[13] Kawachi I, Subramanian SV, Kim D (2008). Social capital and health.

[14] Adler PS, Kwon S-W, Heckscher C (2008). Perspective - professional work: the emergence of collaborative community. Organ Sci.

[15] Hammer A, Arah OA, Dersarkissian M, Thompson CA, Mannion R, Wagner C (2013). The relationship between social capital and quality management systems in European hospitals: a quantitative study. PLoS One.

[16] Ernstmann N, Driller E, Kowalski C, Karbach U, Jung J, Pfaff H, Ommen O (2013). Social capital and quality emphasis: a cross-sectional multicenter study in German hospitals. Int J Healthc Manag.

[17] Ernstmann N, Ommen O, Driller E, Kowalski C, Neumann M, Bartholomeyczik S, Pfaff H (2009). Social capital and risk management in nursing. J Nurs Care Qual.

[18] Shin JI, Lee E (2016). The effect of social capital on job satisfaction and quality of care among hospital nurses in South Korea. J Nurs Manag.

[19] Ansmann L, Wirtz M, Kowalski C, Pfaff H, Visser A, Ernstmann N (2014). The impact of the hospital work environment on social support from physicians in breast cancer care. Patient Educ Couns.

[20] van Bogaert P, Kowalski C, Weeks SM, van Heusden D, Clarke SP (2013). The relationship between nurse practice environment, nurse work characteristics, burnout and job outcome and quality of nursing care: a cross-sectional survey. Int J Nurs Stud.

[21] Murayama H, Fujiwara Y, Kawachi I (2012). Social capital and health: a review of prospective multilevel studies. J Epidemiol.

[22] Gloede TD, Ernstmann N, Baumann W, Groß SE, Ansmann L, Nitzsche A (2015). Fluktuation bei nicht-ärztlichem Personal in onkologischen Schwerpunktpraxen: Sozialkapitalaufbau als Lösungsansatz? [Turnover of Non-medical Staff in Outpatient Oncology Practices: Is Building Social Capital a Solution?]. Gesundheitswesen.

[23] Kowalski C, Ommen O, Driller E, Ernstmann N, Wirtz MA, Köhler T, Pfaff H (2010). Burnout in nurses - the relationship between social capital in hospitals and emotional exhaustion. J Clin Nurs.

[24] Ommen O, Driller E, Köhler T, Kowalski C, Ernstmann N, Neumann M (2009). The relationship between social capital in hospitals and physician job satisfaction. BMC Health Serv Res.

[25] Kowalski C, Driller E, Ernstmann N, Alich S, Karbach U, Ommen O (2010). Associations between emotional exhaustion, social capital, workload, and latitude in decision-making among professionals working with people with disabilities. Res Dev Disabil.

[26] Putnam RD (1995). Bowling alone: america’s declining social capital. J Democr.

[27] Bourdieu P, Richardson JG (1986). The forms of capital. The handbook of theory and research for the sociology of education.

[28] Kwon S-W, Adler PS (2014). Social capital: maturation of a field of research. AMR.

[29] Bauman Z (2001). Community: seeking safety in an insecure world.

[30] Kociatkiewicz J (2014). Liquid organization: Zygmunt Bauman and organization theory.

[31] Moore S, Kawachi I (2017). Twenty years of social capital and health research: a glossary. J Epidemiol Community Health.

[32] Sheingold BH, Sheingold SH (2013). Using a social capital framework to enhance measurement of the nursing work environment. J Nurs Manag.

[33] Kouvonen A, Kivimäki M, Vahtera J, Oksanen T, Elovainio M, Cox T (2006). Psychometric evaluation of a short measure of social capital at work. BMC Public Health.

[34] Oksanen T, Kawachi I, Kouvonen A, Takao S, Suzuki E, Virtanen M (2013). Workplace determinants of social capital: cross-sectional and longitudinal evidence from a Finnish cohort study. PLoS One.

[35] Oksanen T, Kivimäki M, Kawachi I, Subramanian SV, Takao S, Suzuki E (2011). Workplace social capital and all-cause mortality: a prospective cohort study of 28,043 public-sector employees in Finland. Am J Public Health.

[36] Tsuboya T, Tsutsumi A, Kawachi I (2015). Change in psychological distress following change in workplace social capital: results from the panel surveys of the J-HOPE study. Occup Environ Med.

[37] Pfaff H, Pühlhofer F, Brinkmann A, Lütticke J, Nitzsche A, Steffen P (2004). Der Mitarbeiterkennzahlenbogen (MIKE). Kompendium valider Kennzahlen. Kennzahlenhandbuch.

[38] Zeike S, Ansmann L, Lindert L, Samel C, Kowalski C, Pfaff H (2018). Identifying cut-off scores for job demands and job control in nursing professionals: a cross-sectional survey in Germany. BMJ Open.

[39] Pinneau SR (1976). Effects of social support on occupational stresses and strains: a symposium presented at the 84^th^ annual convention of the American Psychological Association.

[40] Zapf D, Bamberg E, Dunckel H, Frese M, Greif S, Mohr G (1983). Dokumentation der Skalen des Forschungsprojekts “Psychischer Streß am Arbeitsplatz - Hemmende und fördernde Bedingungen für humanere Arbeitsplätze”: Schlußbericht für das BMFT.

[41] Susanne Lehner B, Jung J, Stieler-Lorenz B, Nitzsche A, Driller E, Wasem J, Pfaff H (2013). Psychosocial factors in the information and communication technology sector. Manag Decis.

[42] Schaufeli WB, Bakker AB, Salanova M (2006). The measurement of work engagement with a short questionnaire. Educ Psychol Meas.

[43] Büssing A, Perrar KM (1992). Die Messung von burnout: Untersuchung einer deutschen Fassung des Maslach burnout inventory (MBI-D) [measuring burnout: a study of a German version of the Maslach burnout inventory (MBI-D)]. Diagnostica.

[44] Topp CW, Østergaard SD, Søndergaard S, Bech P (2015). The WHO-5 well-being index: a systematic review of the literature. Psychother Psychosom.

[45] Kline RB (2008). Principles and practice of structural equation modeling.

[46] van Bogaert P, Timmermans O, Weeks SM, van Heusden D, Wouters K, Franck E (2014). Nursing unit teams matter: impact of unit-level nurse practice environment, nurse work characteristics, and burnout on nurse reported job outcomes, and quality of care, and patient adverse events - a cross-sectional survey. Int J Nurs Stud.

[47] van Bogaert P, van Heusden D, Timmermans O, Franck E (2014). Nurse work engagement impacts job outcome and nurse-assessed quality of care: model testing with nurse practice environment and nurse work characteristics as predictors. Front Psychol.

[48] Guillemin F, Bombardier C, Beaton D (1993). Cross-cultural adaptation of health-related quality of life measures: literature review and proposed guidelines. J Clin Epidemiol.

[49] Andersen LL, Poulsen OM, Sundstrup E, Brandt M, Jay K, Clausen T (2015). Effect of physical exercise on workplace social capital: cluster randomized controlled trial. Scand J Public Health.

